# Fine Mapping of a Novel *defective glume 1* (*dg1*) Mutant, Which Affects Vegetative and Spikelet Development in Rice

**DOI:** 10.3389/fpls.2017.00486

**Published:** 2017-04-06

**Authors:** Haiping Yu, Banpu Ruan, Zhongwei Wang, Deyong Ren, Yu Zhang, Yujia Leng, Dali Zeng, Jiang Hu, Guangheng Zhang, Li Zhu, Zhenyu Gao, Guang Chen, Longbiao Guo, Wenfu Chen, Qian Qian

**Affiliations:** ^1^Rice Research Institute of Shenyang Agricultural University/Key Laboratory of Northern Japonica Rice Genetics and Breeding, Ministry of Education and Liaoning Province/Key Laboratory of Northeast Rice Biology and Breeding, Ministry of AgricultureShenyang, China; ^2^State Key Laboratory of Rice Biology, China National Rice Research InstituteHangzhou, China

**Keywords:** rice (*Oryza sativa* L.), *dg1* mutant, dwarfism and rolled leaves, leaf angle, BR signaling, glumes, grain size

## Abstract

In cereal crops, vegetative and spikelet development play important roles in grain yield and quality, but the genetic mechanisms that control vegetative and spikelet development remain poorly understood in rice. Here, we identified a new rice mutant, *defective glume 1* (*dg1*) mutant from cultivar Zhonghua11 after ethyl methanesulfonate treatment. The *dg1* mutant displayed the dwarfism with small, rolled leaves, which resulted from smaller cells and more bulliform cells. The *dg1* mutant also had an enlarged leaf angle and defects in brassinosteroid signaling. In the *dg1* mutant, both the rudimentary glume and sterile lemma (glumes) were transformed into lemma-like organ and acquired the lemma identity. Additionally, the *dg1* mutant produced slender grains. Further analysis revealed that *DG1* affects grain size by regulating cell proliferation and expansion. We fine mapped the *dg1* locus to a 31-kb region that includes eight open reading frames. We examined the DNA sequence and expression of these loci, but we were not able to identify the *DG1* gene. Therefore, more work will be needed for cloning and functional analysis of *DG1*, which would contribute to our understanding of the molecular mechanisms behind whole-plant development in rice.

## Introduction

Rice (*Oryza sativa* L.) is one of the most important cereal crops and feeds more than half of the world’s population as a staple food. The vegetative and floral organs are very important agricultural organs that determine grain yield and quality. In eudicots, the typical flowers have four whorls of floral organs: sepals in whorl 1, petals in whorl 2, stamens in whorl 3, and pistils in whorl 4 from the outer to inner whorls. The classical ABCDE model proposes that how A/B/C/D/E class genes act in combination to specify the identity of each organ and affect floral meristem determinacy (Coen and Meyerowitz, 1991; Jeon et al., 2000; Theissen and Saedler, 2001; Nagasawa et al., 2003; Ditta et al., 2004; Yamaguchi and Hirano, 2006; Dreni et al., 2007; Gao et al., 2010). A- and E-function genes are responsible for the sepal formation (Wang et al., 2010; Kobayashi et al., 2012); A-, B- and E-function genes together determine the petal identity (Nagasawa et al., 2003; Prasad and Vijayraghavan, 2003; Yao et al., 2008; Yun et al., 2013); B-, C-, and E-function genes together modulate the stamen development (Kyozuka and Shimamoto, 2002; Yamaguchi and Hirano, 2006; Dreni et al., 2011; Yun et al., 2013); and C- and E-function genes act together to regulate the pistil identity (Coen and Meyerowitz, 1991; Pelaz et al., 2000; Theissen and Saedler, 2001; Ditta et al., 2004; Gao et al., 2010). D-and E-function genes together specify the placenta and ovule identity (Dreni et al., 2007; Gao et al., 2010; Li et al., 2011). This genetic model is applicable to monocot species including rice and maize (Nagasawa et al., 2003; Dreni et al., 2007), except that the non-MADS-box homeotic gene *DROOPINGLEAF* (*DL*) are recruited and mainly determines the pistil identity in rice (Yamaguchi et al., 2004; Yamaguchi and Hirano, 2006; Sang et al., 2012).

Vegetative and reproductive development plays important roles in high yield because plant architecture is the foundation of reproductive development, and the development of spikelet which directly produced seeds determines the final grain quantity and quality. Ongoing genetic research has identified several genes that pleiotropic affect vegetative growth and spikelet development in rice. Rice *Aberrant Panicle Organization 1* (*APO1*), the ortholog of Arabidopsis *Unusual Floral Organ* (*UFO*), encodes an F-box protein and functions in leaf formation, inflorescence shape and the identity of floral organs (Ikeda et al., 2005, 2007; Ikeda-Kawakatsu et al., 2009). The *apo1* mutant produces more leaves, smaller inflorescences, lodicule-like stamens, and extra carpels, suggesting that the mutant affects the identity of floral organs and floral meristem determinacy. *Super Apical Dormant1* (*SAD1*) encodes a Mediator-interacting protein that binds to OsMED4 and controls various aspects of plant development (Duan et al., 2012). The *sad1* mutant exhibited a low number of tillers, severe dwarfism, slender and short leaves, and abnormal floral organs. *Dwarf and Deformed Flower1* (*DDF1*) and *Abnormal Flower and Dwarf1* (*AFD1*) encodes an RNA polymerase I and a DUF640 domain protein, respectively (Li et al., 2015; Ren et al., 2016b). The loss-of-function mutants of *DDF1* and *AFD1* displayed variable defects such as dwarfism, low seed-setting rate, and defective floral organs, suggesting that *DDF1* and *AFD1* are involved in the regulation of cell expansion proliferation, and floral organ identity. More work is required to better understand the molecular mechanism that controls vegetative growth and reproductive development in rice.

In this study, we characterized a new recessive mutant, *defective glume 1* (*dg1*) from an M_2_ population of the cultivar Zhonghua11 (ZH11) treated with ethyl methanesulfonate (EMS). The *dg1* mutant displayed a wide range of defects, including dwarfism, increased leaf angle, small and rolled leaves, bushy stigmas.

Our observations of the elongated glumes (rudimentary glumes and sterile lemmas) with lemma-like cellular patterns in the *dg1* mutant indicated that the rudimentary glume, sterile lemma, and lemma may be homologous organs and *DG1* plays important roles in the determination of floral organ identity. In the *dg1* mutant, we observed slender grains, which were caused by abnormalities in the number and size of the hull (i.e., lemma and palea) cells. Our results also showed that *dg1* mutant was a brassinosteroid (BR)-hypersensitive and *DG1* was involved in regulating BR signaling. Further, we investigated the phenotypes of the *dg1* mutant and fine mapped the *dg1* locus. DNA sequencing and gene expression were used in an attempt to clone the *DG1* gene.

## Materials and Methods

### Plant Materials

The *dg1* mutant of rice (*O. sativa*) was obtained from the EMS treatment of the *japonica* cultivar ZH11. ZH11 plants were used as the wild-type strain for phenotypic observation. The *dg1* mutant was crossed with *indica* cultivars Nanjing 6 (NJ6) and Taichung Native 1 (TN1) and the F_1_ plants were self-pollinated to generate the F_2_ population. Rice plants were cultivated at the experimental paddy in China National Rice Research Institute, Hangzhou from May to October, and in Lingshui, Hainan Province from January to May.

### Identification of *DG1*

The *dg1* mutation was primary mapped in the F_2_ population of a cross between the *dg1* mutant and the *indica* cultivar NJ6, with which six simple sequence repeat (SSR) markers from publicly available rice databases flanking the target region. For fine mapping of the *dg1* mutation, more F_2_ plants were screened and new insertion or deletion (InDel) molecular markers were used. The sequences of primers are listed in Supplementary Table 1.

### Microscopic Examination

Fresh spikelet specimens were acquired from the same position of ZH11 and the *dg1* mutant grown under the same conditions. Specimens were fixed at 4°C overnight in 50% FAA solution (50% ethanol, 10% formaldehyde, 5% glacial acetic acid, and 35% sterile water). Spikelet specimens were placed under vacuumed for about 30 min prior to fixation to ensure the materials were completely immersed in the FAA solution. The fixed samples were then treated with an ethanol gradient (50, 70, 80, 90, 100, 100, and 100%). Spikelet specimens were infiltrated in xylene and embedded in Paraplast Plus (Sigma). Using a microtome (HM340E), samples were cut into approximately 8-μm thick sections and flattened on glass slides coated with poly-lysine. After deparaffinating the samples in a xylene series and dehydrating them with an ethanol series, the samples were stained using 1% safranine and 1% Fast Green, then dehydrated in an ethanol series and cleared in a xylene series. For microscopy, sections were covered with neutral resins at 42°C for 48 h. Optical microscopy was conducted with a Nikon Eclipse 90i microscope. For electron microscopy, fresh spikelet hull specimens were observed using a HITACHI S-3500 scanning electron microscope that had a –30°C cool stage. We examined cell size, number, and area using the measurement tools of the Nikon Eclipse 90i microscope, and six biological repeats were examined for cell size, cell area, and cell number to obtain the final results.

### RNA Extraction and Quantitative Real-Time PCR (qRT-PCR) Analysis

The total RNA was isolated from floral organs and inflorescences of the wild type and *dg1* mutant using the AxyPrep^TM^ total RNA Miniprep Kit (Axygen) according to the manufacturer’s instructions. The first strand of complementary DNA (cDNA) was reverse transcribed from 500 ng total RNA in a 50 uL reaction volume using the ReverTra Ace^®^ quantitative PCR RT Master Mix Kit with gDNA remover (Toyobo) and qRT-PCR was conducted with a CFX96 Touch^TM^ Real-time PCR Detection System and the 2x SsoFast^TM^ EvaGreen^®^ SuperMix (Bio-Rad) to amplify the cDNA of all tested genes. All target genes were normalized to the rice internal control gene *Actin* to detect the relative expression levels. Three biological repeats were conducted to obtain the final results. The primers for the qRT-PCR assays are listed in Supplementary Table 1.

### BR Response Test

Germinated seeds from ZH11 and *dg1* mutant were grown in standard culture solutions of 0, 0.01, 0.1, and 1 uM 24-Epibrassinolide (24-eBL) under darkness for a week, respectively. The angle of leaf inclination was then measured and photographed.

## Results

### Defects in Vegetative Development in the *dg1* Mutant

**FIGURE 1:**
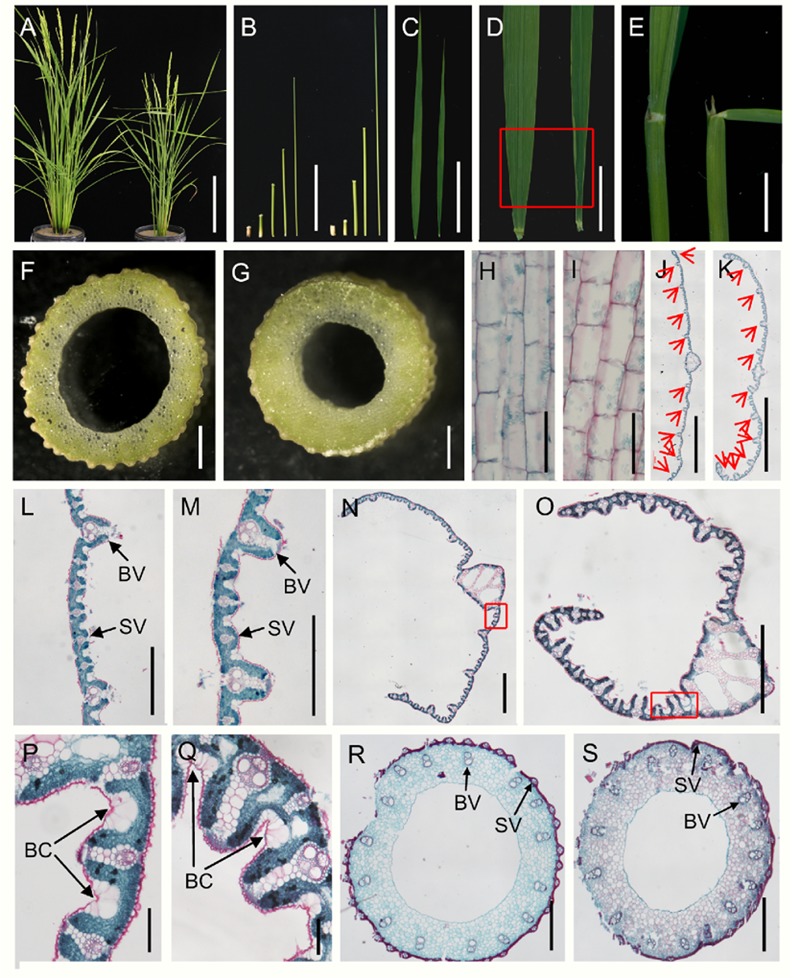
**Morphological comparison of the wild type and *dg1* mutant. (A)** Wild type (left) and *dg1* plants (right) at heading stage. **(B)** Lengths of internodes in the *dg1* mutant (left) and wild type (right); fifth internode of the wild type, fourth internode of the wild type, third internode of the wild type, second internode of the wild type, first internode of the wild type, fifth internode of the *dg1* mutant, fourth internode of the *dg1* mutant, third internode of the *dg1* mutant, second internode of the *dg1* mutant, and first internode of the *dg1* mutant from left to right. **(C)** Comparison of leaf morphology in the wild type (left) and *dg1* mutant (right). **(D)** Base of flag leaves in the wild type (left) and *dg1* mutant (right). **(E)** Comparison of flag leaf angle. **(F)** Culm of the wild type. **(G)** Culm of *dg1* mutant. **(H)** Longitudinal section of the first internode in the wild type. **(I)** Longitudinal section of the first internode in the *dg1* mutant. **(J)** Cross section of the middle part in the wild type leaf. **(K)** Cross section of the middle part in the *dg1* leaf. **(L)** Partial magnification in **(J)**. **(M)** Partial magnification in **(K)**. **(N)** Cross section of the base of the flag leaves in the wild type. **(O)** Cross section of the base of the flag leaves in the *dg1* mutant. **(P)** Magnification of red box region in **(N)**. **(Q)** Magnification of red box region in **(O)**. **(R)** Cross section of the culm in the wild type. **(S)** Cross section of the culm in the the *dg1* mutant. BV, big vascular bundle. SV, small vascular bundle. BC, bulliform cell. Red arrows represent big vascular bundles. Bars = 20 cm in **(A)**; 10 cm in **(B,C)**; 2 cm in **(D)**; 1 cm in **(E)**; 5 mm in **(F,G)**; 100 μm in **(H,I,P,Q)**; 2000 μm in **(J,K)**; 500 μm in **(L,M,R,S)**. 1000 μm in **(N,Q)**.

In the vegetative phase, the *dg1* mutant was shorter and thinner than the wild type (**Figures 1A,B** and Supplementary Figure 1). The first internode was distinctly shorter in the *dg1* mutant, but the second, third, fourth, and fifth internodes showed no differences when compared with the wild type (**Figure 1B** and Supplementary Figure 1). The culms and leaves of *dg1* mutant were thinner than those of the wild type (**Figures 1C,D,F,G** and Supplementary Figure 1) and the base of the flag leaves was rolled (**Figure 1D**). In addition, the leaf angle was enlarged in the *dg1* mutant compared with the wild type; the angle of leaf inclination was increased by 51% in the *dg1* mutant (**Figure 1E** and Supplementary Figure 1). Additionally, the average collar length of the adaxial surfaces in the lamina joint of the *dg1* mutant was increased by 40%, while the average collar length in the abaxial side was indistinguishable from the wild type (Supplementary Figure 1).

We also performed histocytological analysis to study the cell structures. The cells were shorter in the first internodes of the *dg1* mutant (**Figures 1H,I** and Supplementary Figure 1). Analysis of cross sections of the culms and leaves showed that the total cell numbers were reduced and the number of small vascular bundles was markedly decreased in the *dg1* mutant compared with the wild type (**Figures 1J,K,L,M,R,S** and Supplementary Figure 1). However, the number of big vascular bundles remained unchanged between the wild type and the *dg1* mutant (**Figures 1J,K,R,S** and Supplementary Figure 1). These results suggested that *DG1* affects the diameter of culms and the width of leaves mainly by regulating the number of small vascular bundles. In the *dg1* leaves, the number and area of bulliform cells were also increased (**Figures 1N,O,P,Q** and Supplementary Figure 1), resulting in adaxial leaf rolling.

### The *dg1* Mutant is Hypersensitive to BR and Has Altered Expression of BR Response and Biosynthesis Genes

**FIGURE 2:**
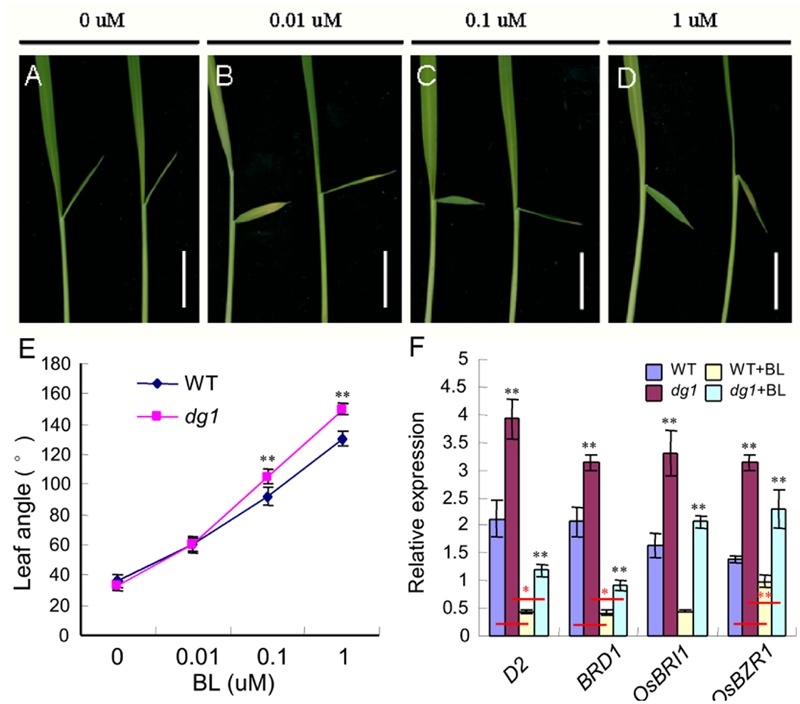
**Morphological response to brassinosteroid (BR) treatment. (A–D)** Phenotype of the wild type (left) and *dg1* mutant (right) after BR treatment. **(E)** Statistical analysis of the leaf angle in the wild type and *dg1* mutant after BR treatment. **(F)** expression analysis of BR biosynthetic and signaling genes. 0, 0.01, 0.1, and 1 μM represent a series of BR concentrations. Total RNA was isolated from the leaves of the untreated wild type, the untreated *dg1* mutant, the wild type after BR treatment and the *dg1* mutant after BR treatment at the seedling stage. WT, wild type. Black asterisk indicates the significant difference of the expression levels of *D2, BRD1, OsBRI1*, and *OsBZR1* between the wild type and *dg1* mutant. Red asterisk indicates the significant difference of the decreased range of expressions levels of *D2, BRD1*, and *OsBZR1* between the wild type group and *dg1* group. Error bars indicate SD. ^∗∗^Significant difference at *P* < 0.01 compared with the wild type by Student’s *t*-test. ^∗^Significant difference at *P* < 0.05 compared with the wild type by Student’s *t*-test. Bars = 0.5 cm.

Brassinosteroid plays a vital role in plant growth and development, especially in leaf morphology and leaf angle (Sakamoto et al., 2013; Zhang et al., 2015). Thus, to test whether the *dg1* mutant has defects in BR signaling, we conducted a BR sensitivity experiment using different concentrations of 24-eBL and measured the degree of leaf inclination. Notably, the *dg1* mutant showed a larger lamina joint angle than that of the wild type after treatment with 0.1 μM 24-eBL (**Figures 2A–E**). This showed that the *dg1* mutant was more hypersensitive to BR than the wild type and suggested that *dg1* mutant may be related to BR signaling. We examined the expressions of related genes on BR response and biosynthesis in the BR-treated and untreated wild type and *dg1* plants by qRT-PCR. In the untreated plants, the expression of the BR response genes *OsBRI1* (*brassinosteroid insensitive 1*) and *OsBZR1* (*Brassinazole-resistant 1*), was higher in the *dg1* mutant than in the wild type (**Figure 2F**). Similarly, the mRNA levels of BR biosynthetic genes *D2 (CYP90D2)* and *BRD1 (BR-deficient dwarf1)* were significantly up-regulated in the *dg1* mutant compared with those of the wild type under normal growth condition (**Figure 2F**). In the treated plants, the expression levels of *OsBRI1, OsBZR1, D2* and *BRD1* were reduced in the wild type and *dg1* mutant.

Compared with the wild type without BR treatment, the expression of *D2, BRD1* and *OsBZR1* in the wild type under BR treatment was reduced by 70, 73, and 23%, respectively. By contrast, the expression of *D2, BRD1* and *OsBZR1* in the *dg1* mutant under BR treatment was reduced by 75, 77, and 31%, respectively. The decreased ranges of expressions of *D2, BRD1* and *OsBZR1* were higher in the *dg1* mutant than that in the wild type under BR treatment, further revealing that the *dg1* mutant was hypersensitive to exogenous BR. These findings indicated that *OsBRI1, OsBZR1, D2* and *BRD1* were modulated by *DG1* under normal growth condition, and confirmed that *DG1* is involved in regulating BR signaling.

### Phenotypic Defects of Spikelet in the *dg1* Mutant

**FIGURE 3:**
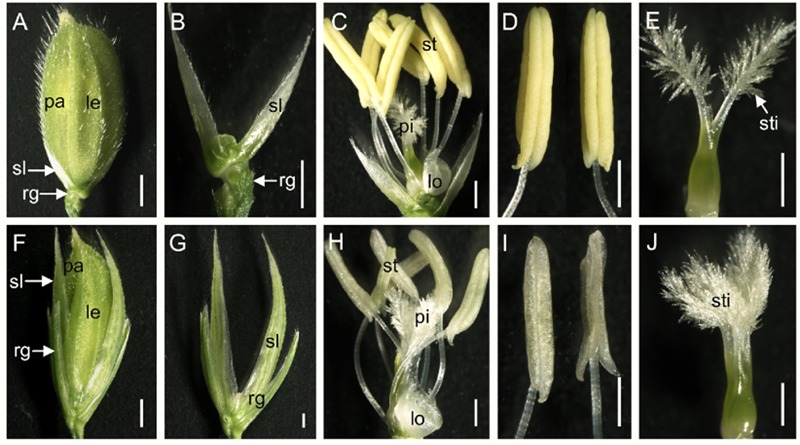
**Phenotypes of the spikelets in the wild type and the *dg1* mutant. (A)** Wild-type spikelet. **(B)** Rudimentary glume and sterile lemma in the wild type. **(C)** Wild type floret. **(D)** Stamen of the wild type floret. **(E)** Pistil of the wild type floret. **(F)** *dg1* spikelet. **(G)** Rudimentary glume and sterile lemma in the *dg1* mutant. **(H)** *dg1* floret. **(I)** Stamen of the *dg1* floret. **(J)** Pistil of the *dg1* floret. rg, rudimentary glume; sl, sterile lemma; le, lemma; pa, palea; sti, stigma; lo, lodicule; st, stamen; pi, pistil. Bars = 1000 μm in **(A,F)**; 500 μm in **(B–E,G–J)**.

The wild type spikelet in rice is composed of two pairs of vestigial glumes: rudimentary glumes and sterile lemmas, and one terminal floret comprising a lemma, a palea, two lodicules, six stamens and a central pistil (**Figures 3A–E**). At heading stage, we found that about 39% spikelets showed longer rudimentary glumes in the *dg1* mutant (**Figures 3A,B,F,G**). The average size of wild type rudimentary glumes was about 0.5 mm length, while the size of rudimentary glumes was variable, from 1.1 to 4.0 mm in length in the 39% spikelets of the *dg1* mutant (Supplementary Table 2). About 94% spikelets of the *dg1* mutant produced larger sterile lemmas that were similar to the lemmas of the wild type or *dg1* mutant (**Figures 3A,B,F,G**). The length of the *dg1* sterile lemmas was varied from 6.0 to 7.9 mm (Supplementary Table 2).

**FIGURE 4:**
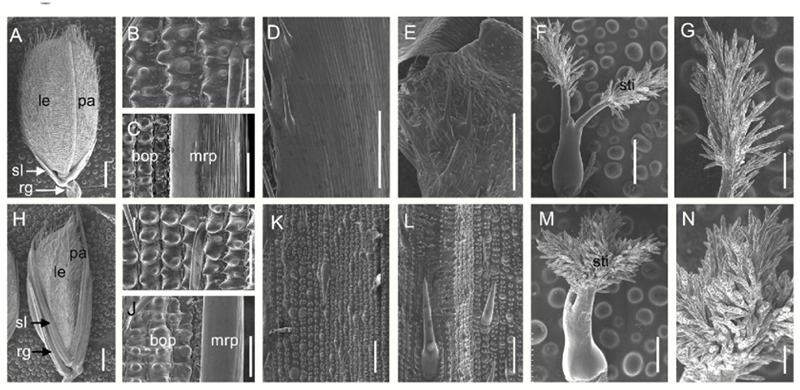
**Scanning electron microscopy (SEM) analysis of floral organs in the wild type and the *dg1* mutant at heading stage. (A)** Wild-type spikelet. **(B)** Epidermal surface of the lemma in the wild type. **(C)** Epidermal surface of the palea in the wild type. **(D)** Epidermal surface of the sterile lemma in the wild type. **(E)** Epidermal surface of the rudimentary glume in the wild type. **(F)** Pistil in the wild-type floret. **(G)** Stigma of pistil in the wild-type floret. **(H)** *dg1* spikelet. **(I)** Epidermal surface of the lemma in the *dg1* mutant. **(J)** Epidermal surface of the palea in the *dg1* mutant. **(K)** Epidermal surface of sterile lemma in the *dg1* mutant. **(L)** Epidermal surface of the rudimentary glume in the *dg1* mutant. **(M)** Pistil in the *dg1* floret. **(N)** Stigma of pistil in the *dg1* floret. rg, rudimentary glume; sl, sterile lemma; le, lemma; pa, palea; bop, body of palea; mrp, marginal region of palea; sti, stigma. Bars = 1000 μm in **(A,H)**; 500 μm in **(F,M)**; 200 μm in **(G,N)**; and 100 μm in **(B–E,I–L)**.

We next investigated the floral organs of four whorls in the *dg1* flowers. Interestingly, the *dg1* mutant showed slightly whiter and smaller stamens, and highly bushy stigmas compared with the wild type (**Figures 3C,D,E,H,I,J**). However, the number of floral organs was not changed in the *dg1* mutant (**Figures 3A,C,F,H**). To investigate the cellular morphology, we performed paraffin section and scanning electron microscopy (SEM). In the wild type, the lemma had five vascular bundles and two unique inward hook-like structures (Supplementary Figures 2A,C). The palea had three vascular bundles and exhibited a uniquely smooth epidermis that lacked outer silicified epicuticular cells (**Figures 4A–C** and Supplementary Figures 2A,C). The sterile lemma developed one vascular bundle (Supplementary Figure 2D) and had a smooth epidermis with regularly arranged cells and rare trichomes (**Figures 4A,D**). The epidermis of the rudimentary glumes displayed irregularly arranged cells and bore numerous small protrusions and trichomes (**Figures 4A,E**). No obvious vascular bundles were observed in the rudimentary glumes of the wild type (Supplementary Figure 2E). We also found that the stigmas of the wild-type pistil were sparse and the pistils had normal ovaries (**Figures 4F,G** and Supplementary Figures 2B,F,G). By contrast, the sterile lemmas and rudimentary glumes were elongated in the *dg1* mutant (**Figures 4H–J**). The sterile lemmas and rudimentary glumes had more vascular bundles, big protrusions and trichomes, and inward hook-like structures (**Figures 4K,L** and Supplementary Figures 2H–K), which was similar with that of the lemma in the wild type (**Figure 4B** and Supplementary Figures 2A,C). In addition, the *dg1* mutant displayed denser stigmas and an abnormal ovary (**Figures 4M,N** and Supplementary Figures 2B,G).

**FIGURE 5:**
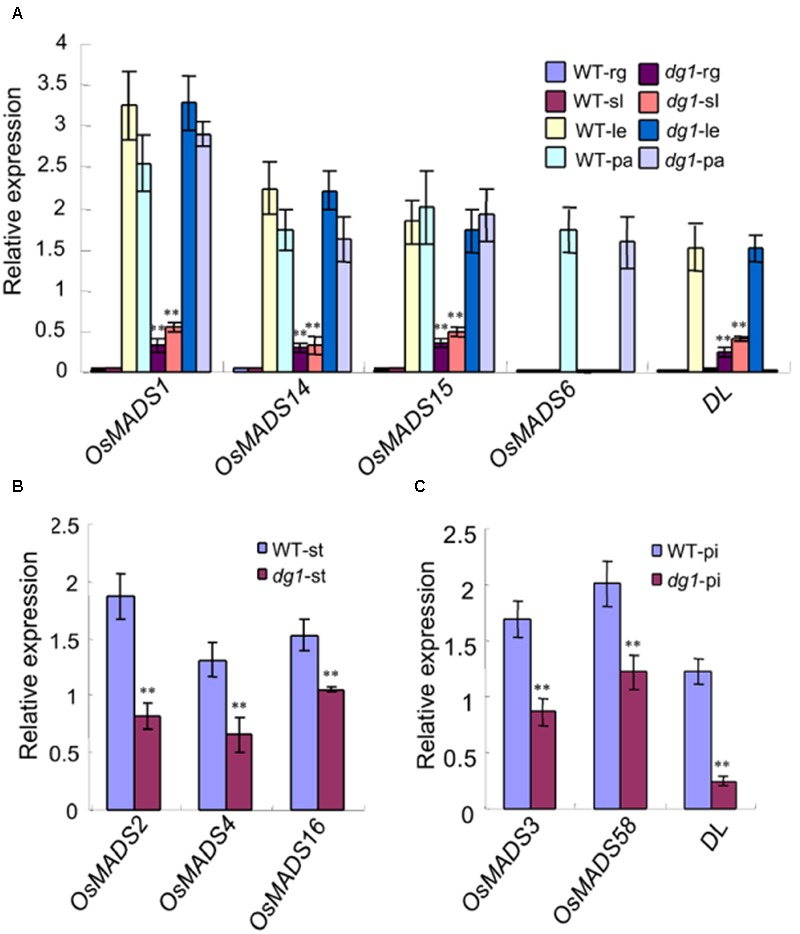
**Expression analysis of genes determining floral organ identity in the wild type and *dg1* mutant. rg, rudimentary glume; sl, sterile lemma; le, lemma; pa, palea; st, stamen; pi, pistil. (A)** The expression of genes in the rudimentary glume and sterile lemma. **(B)** The expression of genes in the stamen. **(C)** The expression of genes in the pistil. Total RNA was isolated from spikelets at heading stage. WT, wild type. Error bars indicate SD. ^∗∗^Significant difference at *P* < 0.01 compared with the wild type by Student’s *t*-test.

To clarify the identities of the floral organs, we examined the expression of related marker genes in the *dg1* spikelets. The expressions of *OsMADS1, OsMADS14, OsMADS15*, and *DROOPING LEAF* (*DL*) was detected in the sterile lemmas and rudimentary glumes of the *dg1* spikelets, implying that the sterile lemmas and rudimentary glumes were converted to lemma-like organs (**Figure 5A**). No *OsMADS6* transcript was found in the sterile lemmas and rudimentary glumes of the wild type and *dg1* spikelets, suggesting that the sterile lemmas and rudimentary glumes did not acquire the palea identity (**Figure 5A**). The expression of *OsMADS2, OsMADS4*, and *OsMADS16* was lower in the *dg1* stamens (**Figure 5B**), and the expression of *OsMADS3, OsMADS58*, and *DL* was lower in the *dg1* pistils (**Figure 5C**), which was consistent with the phenotype defects in the *dg1* mutant. These results revealed that the glumes of the *dg1* mutant partly acquired the lemma identity, and the identities of stamens and pistils were altered.

### Abnormal Early Spikelet Development in the *dg1* Mutant

**FIGURE 6:**
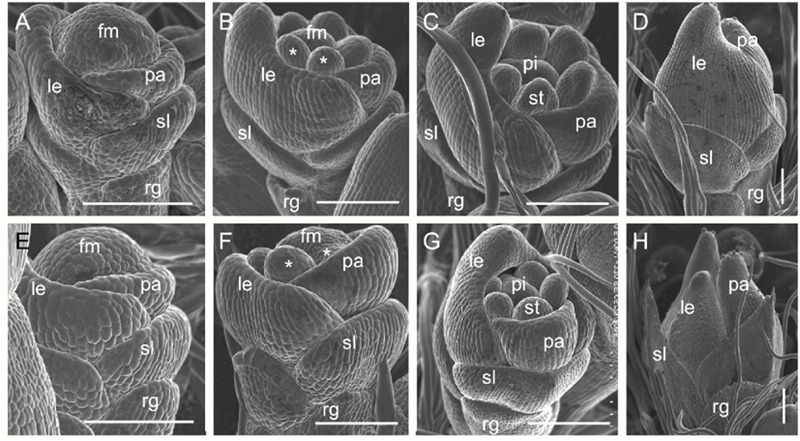
**Early spikelet development in the wild type and the *dg1* mutant. (A–D)** Wild-type spikelet. **(A)** Sp4, **(B)** Sp5-6, **(C)** Sp7, **(D)** Sp8. **(E–H)** *dg1* spikelet. **(E)** Sp4, **(F)** Sp5-6, **(G)** Sp7, **(H)** Sp8. fm, floral meristem; le, lemma; pa, palea; st, stamen; pi, pistil, rg, rudimentary glume; sl, sterile lemma. Bars = 100 μm.

We examined spikelet development in the *dg1* mutant by SEM. The wild-type spikelets formed sterile lemma and rudimentary glume primordia, and the palea and lemma primordia were growing at the spikelet 4 stage (Sp4) (**Figure 6A**). At the Sp5 and Sp6 stages, six stamen primordia were observed in the wild-type spikelet and the growth of the stamen primordium on the lemma side was delayed (**Figure 6B**). Also at this stage the rudimentary glume ceased growing and the sterile lemma continued growing (**Figures 6B,C**). During the Sp7 and Sp8 stages in the wild-type spikelet, the pistil primordium was observed and the sterile lemma was further enlarged and was much longer than the rudimentary glume (**Figures 6C,D**). By contrast, no obvious differences were detected in the rudimentary glume and sterile lemma of the *dg1* spikelets at the Sp4–Sp6 stages (**Figures 6E,F**). At the Sp7 and Sp8 stages, the rudimentary glume appeared normal in the *dg1* spikelets, while the sterile lemma was longer (**Figures 6G,H**). We concluded that the *dg1* glumes continue growing after the Sp8 stage, thus producing further elongated glumes at heading stage, and the rudimentary glume develops at later stages. Additionally, the development of the *dg1* stamens was not synchronous and other floral organs appeared normal in the *dg1* florets. These results revealed that the enlargement of glumes was strongly affected and the development of stamens was disrupted in the *dg1* mutant.

### The *dg1* Mutant Has Reduced Grain Yield

**FIGURE 7:**
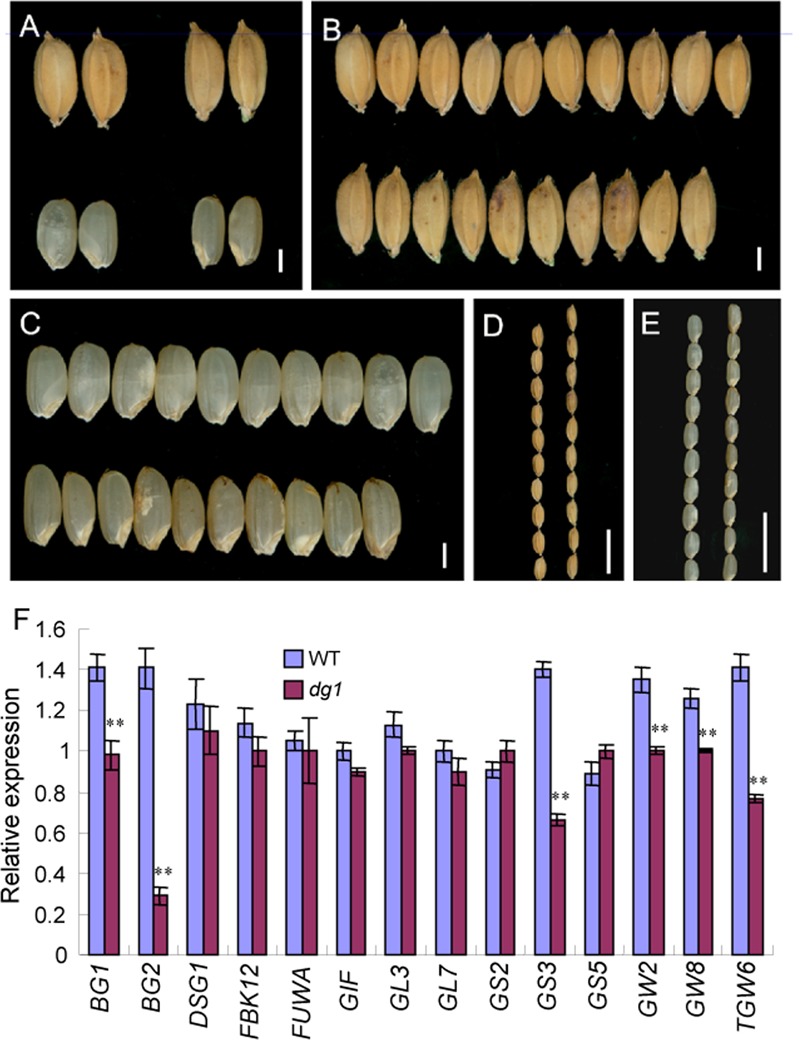
**Phenotypic observations of grain and expression analysis of related genes controlling grain size in the wild type and *dg1* mutant. (A)** Grains phenotype of the wild type (left) and *dg1* mutant (right). **(B)** Grains of the wild type (upper) and *dg1* mutant (lower). **(C)** Brown rice of the wild type (upper) and *dg1* mutant (lower). **(D)** Grains of the wild type (left) and *dg1* mutant (right). **(E)** Brown rice of the wild type (left) and *dg1* mutant (right). **(F)** Expression analysis of related genes controlling grain size in the wild type and *dg1* mutant. Total RNA was isolated from the endosperms at 5 days after fertilization. Bars = 2000 μm in **(A–C)**; 1 cm in **(D, E)**. Error bars indicate SD. ^∗∗^Significant difference at *P* < 0.01 compared with the wild type by Student’s *t*-test.

At maturity, the *dg1* mutant had shorter and smaller panicles (Supplementary Figure 3). Compared with the wild type, the panicles were 33% shorter in the *dg1* mutant (Supplementary Figure 3). The number of spikelets from the *dg1* mutant was only 66% of that of the wild type (Supplementary Figure 3). The pollen viability of the stamens was 52% and the seed-setting rate was 45% in the *dg1* mutant (Supplementary Figures 3, 4), compared with 96 and 85% in the wild type, respectively (Supplementary Figures 3, 4). This suggested that the low seed-setting rate in the *dg1* mutant may be due to defective pollen grains. The wild-type grains and brown rice averaged 7.1 and 5.4 mm long, and 3.5 and 3.0 mm wide, respectively (**Figures 7A–E** and Supplementary Figure 3). The *dg1* grains were longer and thinner than those of the wild type (**Figures 7A,D,E** and Supplementary Figure 3). The *dg1* grains and brown rice averaged 7.9 and 5.6 mm long and 3.3 and 2.8 mm wide, respectively (Supplementary Figure 3). Compared with the wild type, the 1,000-grain weight was not affected in the *dg1* mutant, but the weight of 1,000-brown rice from the *dg1* mutant was decreased markedly (Supplementary Figure 3), suggesting that the proportion of hulls in the *dg1* grains was higher than that of the wild type, which supports the ideal that the hulls can affect grain yield. We also examined the expression of genes involved in the regulation of grain size. Compared with the wild type, *BG1, BG2, GS3, GW2, GW8*, and *TGW6* were down-regulated in the *dg1* mutant (**Figure 7F**). These data supported the phenotypic observations and the ideal that *DG1* may affect grain size by negatively regulating the related genes expression.

**FIGURE 8:**
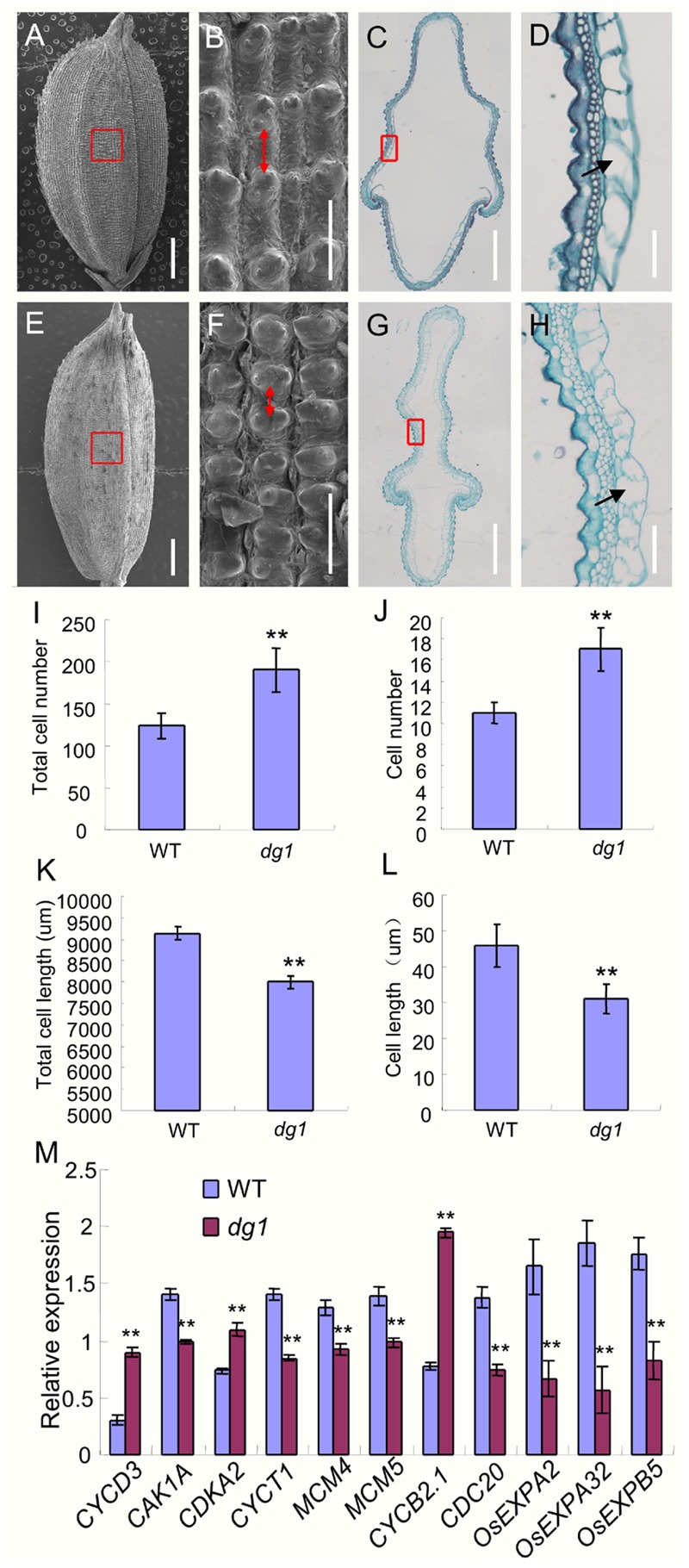
**Histocytological analysis of grain hull in the wild type and *dg1* mutant. (A)** Grain of the wild type. **(B)** Red box region in **(A)**. **(C)** Cross section of the hull in the wild type. **(D)** Partial magnification of red box region in **(C)**. **(E)** *dg1* grain. **(F)** Red box region in **(E)**. **(G)** Cross section of the hull in the *dg1* mutant. **(H)** Partial magnification of red box region in **(G)**. **(I)** Total cell number along the longitudinal axis of the lemma. **(J)** Cell number per millimeter along the longitudinal axis of the lemma. **(K)** Total cell length in the middle part of the hull. **(L)** Cell length. **(M)** Expression analysis of cell cycle and expansion-related genes in panicles of the wild type and *dg1* mutant. Red arrows represent cell length in the epidermal surface of the hull. Black arrows represent cell length in the cross section of the hull. Total RNA was isolated from the spikelets at the heading stage. Error bars indicate SD. ^∗∗^Significant difference at *P* < 0.01 compared with the wild type by Student’s *t*-test.

To investigate the abnormal grains in the *dg1* mutant at the cellular level, we performed histocytological analysis, including SEM and paraffin section. The observations of the outer epidermal cells of the lemmas showed that the cells were compressed longitudinally in the *dg1* mutant (**Figures 8A,B,E,F**). However, the total cell number and the number of cells per millimeter along the longitudinal axis of the lemmas from the *dg1* mutant were significantly increased compared to the wild type (**Figures 8B,F,I,J**), resulting in longer grains in the *dg1* mutant. Cross sections of central parts of the hulls revealed that the *dg1* spikelet contained shorter length of total cells and smaller cells than the corresponding region in the wild type (**Figures 8C,D,G,H,K,L**); this differences likely were responsible for the slender grains observed in the *dg1* mutant. Taken together, our results implied that *DG1* controls grain size by regulating cell number and size.

Because cell number and size were altered in the *dg1* mutant, we next investigated the expression of several genes that regulate cell cycle and cell expansion in rice. The expressions of eight cell cycle-related genes was altered. Compared with the wild type, *CYCD3, CDKA2*, and *CYCB2.1* were up-regulated in the *dg1* mutant, and *CAK1A, CYCT1, MCM4, MCM5*, and *CDC20* were down-regulated in the *dg1* mutant (**Figure 8M**). The expression of three cell expansion-related genes (*OsEXPA2, OsEXPA32*, and *OsEXPB5*) was reduced in the *dg1* mutant compared to the wild type (**Figure 8M**). These results were consistent with the phenotypic observations and implied that *DG1* mainly affects grain size by regulating cell proliferation and cell expansion.

### Genetic Analysis of the *dg1* Mutant

To survey whether the *dg1* mutant phenotype is controlled by multiple genes or a single gene and whether it is dominant or recessive, we performed the reciprocal crosses between the *dg1* mutant and two *indica* cultivars, NJ6 and TN1 to obtain F_1_ and F_2_ plants (**Table 1**). The F_1_ offspring of all combinations showed a normal phenotype (**Table 1**), indicating that the *dg1* mutation is recessive. In the four F_2_ populations, the segregation ratio of the wild type to *dg1* phenotype was 3:1 (**Table 1**), indicating that the mutated phenotype is controlled by a single, recessive nuclear gene.

**Table 1:** Genetic analysis of the phenotype of the *dg1* mutant.

		F_2_				
	Phenotype of F_1_	No. of wild type plants	No. of *dg1* plants	Total plants	*X*^2^ (3:1)	*P*-value
*dg1*/NJ06	Normal	1612	518	2130	0.526	0.468
NJ06/*dg1*	Normal	1165	400	1565	0.261	0.610
*dg1*/TN1	Normal	1209	381	1590	0.913	0.339
TN1/*dg1*	Normal	961	342	1303	1.081	0.299

### Fine Mapping and Candidate Genes Analysis of *DG1*

**FIGURE 9:**
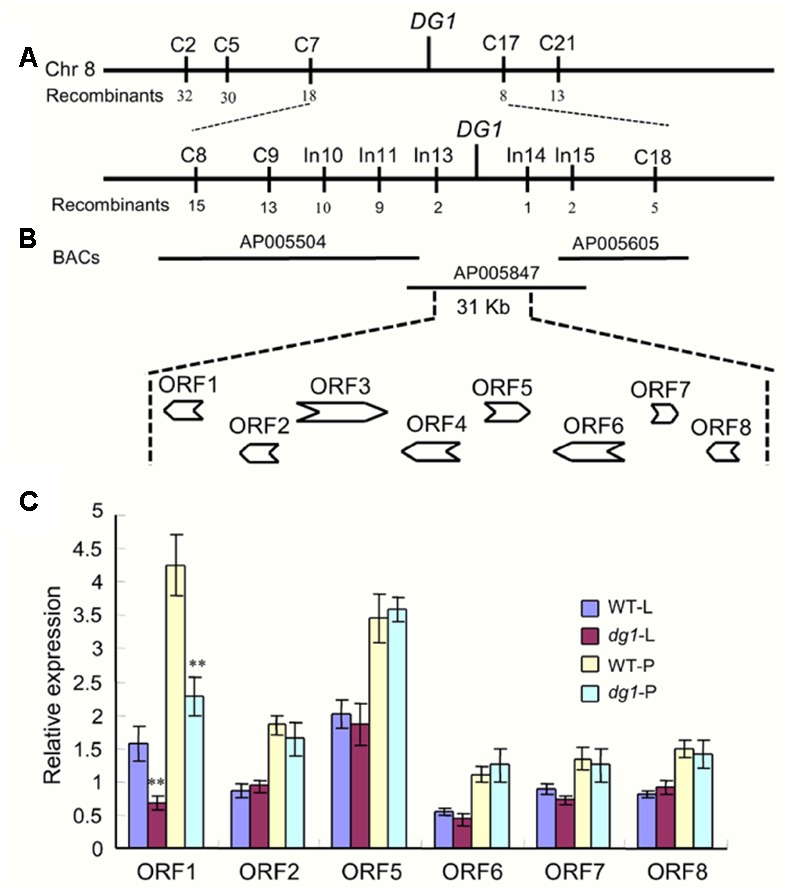
**Fine mapping of the *DG1* gene and analysis of candidate genes. (A,B)** Fine mapping of the *DG1* gene. **(C)** Expression analysis of candidate genes. WT-L, leaf of the wild type at heading stage; *dg1*-L, leaf of the *dg1* mutant at heading stage; WT-P, panicles of the wild type at heading stage; *dg1*-P, panicles of the *dg1* mutant at heading stage. Error bars indicate SD. ^∗∗^Significant difference at *P* < 0.01 compared with the wild type by Student’s *t*-test.

To map the locus responsible for the *dg1* mutant phenotype, we selected the cross of *dg1* mutant and NJ6. We then tested 312 pairs of SSR primers evenly distributed on the 12 rice chromosomes and found that 165 of these markers showed polymorphisms between the two parental lines. Using the polymorphic markers, we screened DNA pools from 15 wild type individuals and 15 recessive *dg1* individuals and compared the pools with the parental lines. Two markers, C2 and C21 on chromosome 8, had the same amplified fragment length in the recessive DNA pool and the *dg1* mutant, and the fragment length differed from that of the wild-type DNA pool. Using this small F_2_ population (70 plants), the mutated locus was primarily mapped to chromosome 8 between markers C2 and C21 (**Figure 9A**).

To further access the *dg1* locus, we designed a large number of SSR and InDel markers flanking C2 and C21. The markers C5, C7, C8, C9, C17, C18, In10, In11, In13, In14, and In15 exhibited polymorphisms between the two parents (**Figure 9A**). Using 518 recessive individuals, the *DG1* gene was finally delimited between InDel markers In13 and In14, an approximately 31-kb physical distance in *Nipponbare* (**Figure 9B**). In the 31-kb target region, there were eight predicted opening reading frames (ORFs) (**Figure 9B**). ORF1 encodes an acyl CoA binding protein that was reported to be related to plant growth and stress tolerance. ORF2 encodes a ribosomal protein and ORF3 and ORF4 encode retrotransposon proteins. ORF5 encodes an expression protein and ORF6 encodes a nuf2 family protein. ORF7 and ORF8 encode an Mps one binder kinase activator-like 1A and a DNA polymerase, respectively. We next sequenced all the annotated genes, but no nucleotide changes were detected between the wild type and *dg1* mutant. We also investigated the expression of all annotated genes in the leaf and panicles by qRT-PCR. Compared with the wild type, only *LOC_Os08g06550* (ORF1) showed a lower level of expression in the *dg1* mutant; no differences were detected in the other genes between the wild type and *dg1* mutant (**Figure 9C**). More work is needed to identify the gene responsible for the *dg1* mutant phenotype.

### The Expression of Genes Related to Floral Development in the *dg1* Mutant

**FIGURE 10:**
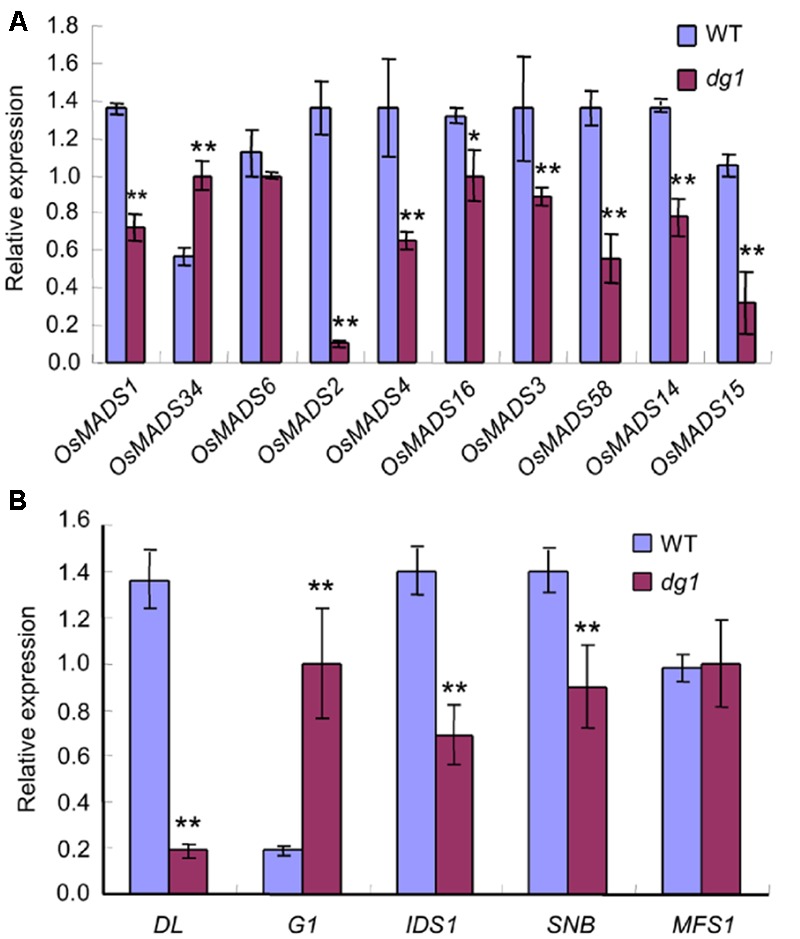
**Expression analysis of related floral development genes. (A)** The expression of MADS-box genes. **(B)** The expression of other related genes. The total RNA was isolated from young panicles at the heading stage. ^∗∗^Significant difference at *P* < 0.01 compared with the wild type by Student’s *t*-test.

Since the *dg1* mutant has defects in spikelet development, we examined whether *DG1* is involved in the regulation of genes associated with floral development in rice. We detected several MADS-box and non-MADS-box genes including A-class genes (*OsMADS14*, and *OsMADS15*), B-class genes (*OsMADS2, OsMADS4*, and *OsMADS16*), C-class genes (*OsMADS3* and *OsMADS58*), E-class genes (*OsMADS1, OsMADS6*, and *OsMADS34*), and non-MADS-box genes (*DL, G1, SNB, IDS1*, and *MFS1*). Except for *OsMADS6* and *OsMADS34*, most of the tested MADS-box genes showed lower expression levels in the *dg1* mutant (**Figure 10A**). Lower expression of *OsMADS1, OsMADS14*, and *OsMADS15* may be attributed to abnormal heading and short panicles in the *dg1* mutant. Abnormal expression of *OsMADS2, OsMADS4, OsMADS16, OsMADS3, OsMADS58*, and *DL* likely caused the defective stamens and pistils in the *dg1* mutant.

We also observed more abundant transcripts of *G1* and *OsMADS34* which restrained the elongation of glumes (rudimentary glume and sterile lemma) (**Figure 10B**) and fewer transcripts of *SNB* and *IDS1* which positively regulated fate of glumes in the *dg1* mutant (**Figure 10B**). These results supported the phenotypic observations and suggested that *DG1* negatively regulated the identities of glumes, resembling the functions of *G1* and *OsMADS34*. Taken together, our findings revealed pleiotropic roles of *DG1* in the floral development and *DG1* regulated the expression of these genes associated with floral development.

## Discussion

Many rice mutants associated with the vegetative development and floral organ identity have been obtained such as *apo1, sad1, opb, afd1*, and *ddf1* (Ikeda et al., 2007; Horigome et al., 2009; Duan et al., 2012; Ikeda-Kawakatsu et al., 2012; Li et al., 2015; Ren et al., 2016b). However, our understanding of vegetative and reproductive development in rice remains limited, and the discovery of more rice mutants will further our knowledge of the regulatory mechanism behind these processes. The *apo1* mutant rapidly produced more leaves than wild type, the stamens were transformed into lodicules, and extra pistils emerged (Ikeda et al., 2007). The *sad1* mutant exhibited a variety of defects including few tillers, severe dwarfism, poor root, and abnormal floral organs (Li et al., 2015). In the *opb* mutant, leaf blades were malformed and the sheath cells partly invaded the blade region. Additionally, lateral growth of the lemma and palea was suppressed and the resulting structure was unable to enclose the inner floral organs (Horigome et al., 2009). Dwarfism occurred in the *afd1* and *ddf1* mutants due to defects in cell proliferation and expansion and the lodicules and stamens were transformed into glume-like organs (Duan et al., 2012; Ren et al., 2016b). The genes responsible for these phenotypes are expressed in all tissues and organs, suggesting that these genes are not tissue-specific and play important roles in the regulation of whole-plant development. In our study, the *dg1* mutant had defects in plant height, leaf morphology, and leaf angle. The *dg1* mutant had slender culms with small and semi-rolled leaves. Further analysis revealed that these defects could attributed to abnormal cell number and cell size in those organs, including fewer vascular bundles, smaller cells, and more bulliform cells.

In our study, the *dg1* mutant had an enlarged leaf angle and BR was a key regulator of leaf angle (Sakamoto et al., 2013; Chen et al., 2015; Zhang et al., 2015). The 24-eBL treatments at concentrations over 0.1 μM induced a lamina joint angle of above 90° in the wild type and *dg1* mutant. However, the *dg1* mutant produced a larger leaf angle than that of the wild type at 24-eBL treatment of 0.1 and 1 μM, and no differences were observed between the wild type and *dg1* mutant at 24-eBL treatment of 0 and 0.01 μM. The expression of BR response and biosynthetic genes *OsBRI1, OsBZR1, CYP90D2*, and *BRD1* were markedly increased in the *dg1* mutant compared with the wild type under normal growth conditions and following BR treatment. However, the decreased ranges of expressions of *D2, BRD1*, and *OsBZR1* were higher in the *dg1* mutant than that in the wild type under BR treatment. These findings suggested that the *dg1* mutant was hypersensitive to BR and may be had defects in BR signaling.

The glumes (i.e., rudimentary glume and sterile lemma) are a unique structure in grass spikelets (Zanis, 2007; Li et al., 2009; Ren et al., 2013, 2016a) and the *dg1* mutation also affected the development of glumes. In the *dg1* mutant, some spikelets exhibited larger rudimentary glumes and sterile lemmas. The rudimentary glume and sterile lemma in the *dg1* mutant exhibited similar protrusions, trichomes, and cell layers, resembling the wild-type lemma. Also, in the *dg1* glumes, *OsMADS1, OsMADS14, OsMADS15*, and *DL* were expressed ectopically in the glumes, but no *OsMADS6* transcript was detected. These results revealed that the glumes of *dg1* mutant partly acquired the lemma identity and *DG1* may be an important regulator of the glume identity.

There are two prevailing hypotheses on the origin and evolution of the glumes. One suggests that the rudimentary glume and sterile lemma are severely reduced bract-like organs (Schmidt and Ambrose, 1998; Terrell et al., 2001; Hong et al., 2010; Ren et al., 2013). The other suggests that the sterile lemma may be derived from a morphological modification of the remaining lemma (Yoshida et al., 2009; Kobayashi et al., 2010; Ren et al., 2016a). *FRIZZY PANICLE* (*FZP*), *SUPERNUMERARY BRACT* (*SNB*), *Oryza sativa INDETERMINATE SPIKELET1* (*OsIDS1*), and *MULTI-FLORET SPIKELET1* (*MFS1*) encode APETALA2/ethyleneresponsive (AP2/ERF) proteins that determine the identity of sterile lemma and/or the rudimentary glume. Loss of function of *FZP* and *SNB* resulted in extra rudimentary glumes, but no sterile lemmas were found in the corresponding position (Komatsu et al., 2003; Lee et al., 2007). A mutation of *OsIDS1* and *MFS1* caused the sterile lemmas to be converted into bract-like organs resembling the rudimentary glumes. The bract-like glume organs of maize and wheat are severely reduced and equivalent to the rudimentary glume in rice, and are similar to the lemma in size and structure (Kellogg, 2001; Yoshida et al., 2009; Hong et al., 2010). The sterile lemmas were elongated and partly acquired the lemma identity in the *dg1, g1*/*ele, osmads34*, and *eg1* mutants (Li et al., 2009; Yoshida et al., 2009; Hong et al., 2010; Gao et al., 2010; Lin et al., 2014; Ren et al., 2016a), which supports the second hypothesis. Therefore, these findings reveal that the rudimentary glume, sterile lemma and the lemma may have been homologous organs during evolution. We also observed slender grains in the *dg1* mutant. Histocytological observation found that the number and size of hull cells were altered in the *dg1* mutant. The qRT-PCR analyses showed that *DG1* affected the expression of cell cycle and expansion related genes, indicating that *DG1* influences grain size by regulating cell proliferation and expansion.

Using the SSR and InDel markers, we mapped the *DG1* locus between InDel markers In13 and In14 on chromosome 8 within a 31-kb physical region. This region contains eight annotated genes, including an acyl CoA binding protein. Although the acyl CoA binding proteins have been associated with floral development, no differences were detected in the DNA sequences between the wild type and *dg1* mutant. However, the expression level of *LOC_Os08g06550* (predicted acyl CoA binding protein) was dramatically decreased in the *dg1* mutant. We also investigated other annotated genes, and the DNA sequences and transcripts levels were not altered between the wild type and *dg1* mutant. Thus, *LOC_Os08g06550* may contribute to the *dg1* mutant phenotypes. However, these findings did not provide valid evidences to identify the *DG1* gene. Other factors besides DNA sequence changes, such as epigenetic alterations can lead to altered phenotypes. A previous study showed that *FERTILIZATION-INDEPENDENT ENDOSPERM 1* (*FIE1*) repressed the expression of the H3K27me3-mediated gene and interacted with rice enhancer of Zeste homologs (Zhang et al., 2012). The *fie1* allele had no nucleotide sequence changes but was hypomethylated in the promoter region of *FIE1*, and *fie1* mutant had dwarfism and various floral defects. Thus, more work is needed to isolate the target gene, such as the analysis of promoter regions and untranslational region sequences of the candidate gene, intergenic enhancers or repressors, and epigenetic regulation in the *dg1* mutant (Luo et al., 2008).

Taken together, cloning and functional analysis of the *DG1* gene would facilitate a better understanding of the molecular mechanisms involved in vegetative and spikelet development in rice, and may provide a new opportunity to improve rice grain yield. Additionally, the *dg1* mutant is an ideal material for further studies of BR signaling.

## Author Contributions

Experimental design: QQ, DR, and WC; Experiments: HY, DR, BR, ZW, YZ, YL, JH, and LZ; Data analysis: DZ, GZ, ZG, GC, and LG; Manuscript preparation: DR and QQ.

## Conflict of Interest Statement

The authors declare that the research was conducted in the absence of any commercial or financial relationships that could be construed as a potential conflict of interest.
