# Supplementary material for: Fine Mapping of a Novel defective glume 1 (dg1) Mutant, Which Affects Vegetative and Spikelet Development in Rice
Source: Front Plant Sci. 2017 Apr 6;8:486. doi: 10.3389/fpls.2017.00486 (PMC5382164; doi:10.3389/fpls.2017.00486)
Supplement: Supplementary file 1 [file Data_Sheet_1.pdf]

SUPPLEMENTAL FIGURES

Supplemental Figure 1

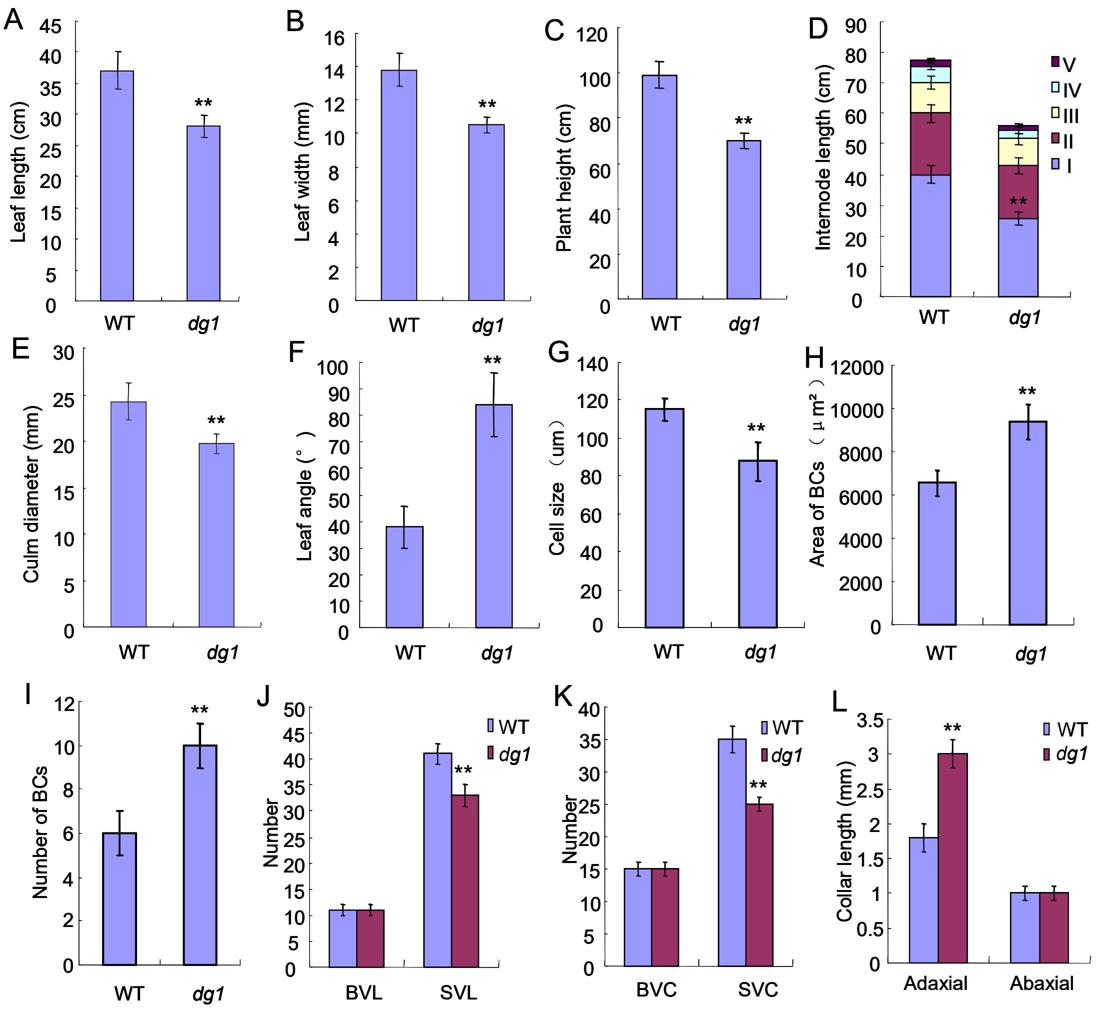

Supplemental Figure 1. Investigation of related agronomic traits in the wild type and *dg1* mutant. A, leaf length. B, leaf width. C, plant height. D, internode length. E, culm diameter. F, leaf angle. G, cell size of the 1st internode. H, area of bulliform cells in leaf. I, number of bulliform cells in leaf. J, number of vascular bundles in leaf. K, number of vascular bundles in culm. L, collar length. BCs, bulliform cells; BVL, big vascular bundles of leaf; SVL, small vascular bundles of leaf; BVC, big vascular bundles of culm; SVC, small vascular bundles of culm. Error bars indicate SD. **Significant difference at $P<0.01$ compared with the wild type by Student's t-test.

Supplemental Figure 2

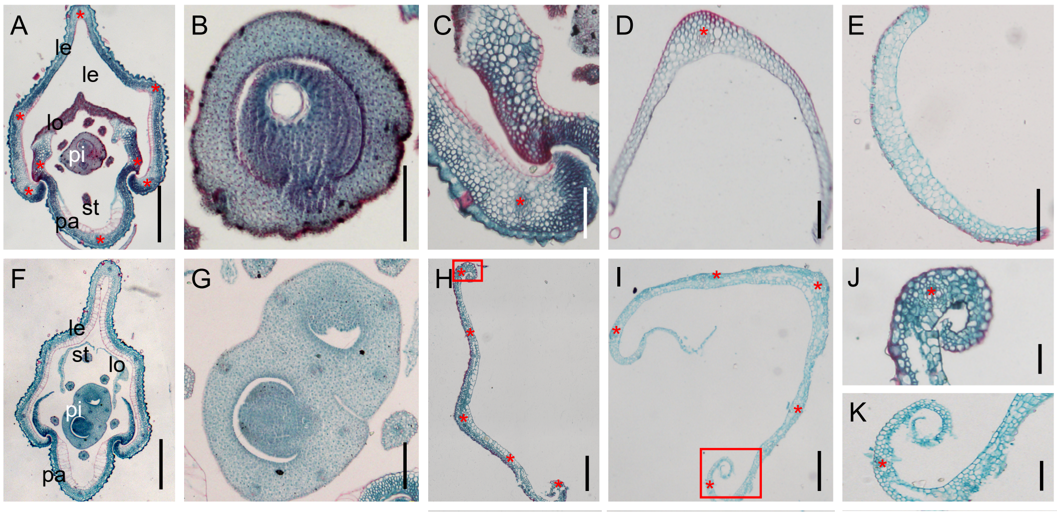

Supplemental Figure 2. Paraffin section analysis in the wild type and *dg1* mutant at heading stage. A, wild type floret; B, wild type pistil; C, hook-like structure of the wild type lemma; D, sterile lemma of the wild type; E, rudimentary glume of the wild type; F, *dg1* floret; G, *dg1* pistil; H, sterile lemma of the *dg1* mutant; I, rudimentary glume of the *dg1* mutant; J, partial magnification of red box region in H; K, partial magnification of red box region in I. le, lemma; pa, palea; lo, lodicule; st, stamen; pi, pistil. Red box represents hook-like structure. Asterisk represents vascular bundle. Bars = 100 μm in A-C, E-G, J and K, 200 um in D, H and I.

Supplemental Figure 3

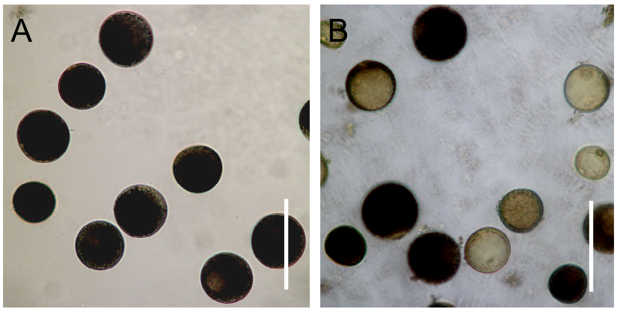

Supplemental Figure 3. Investigation of pollen viability in the wild type and *dg1* mutant. A, pollen viability in the wild type. B, pollen viability in the *dg1* mutant. Bars = 100 μm

Supplemental Figure 4

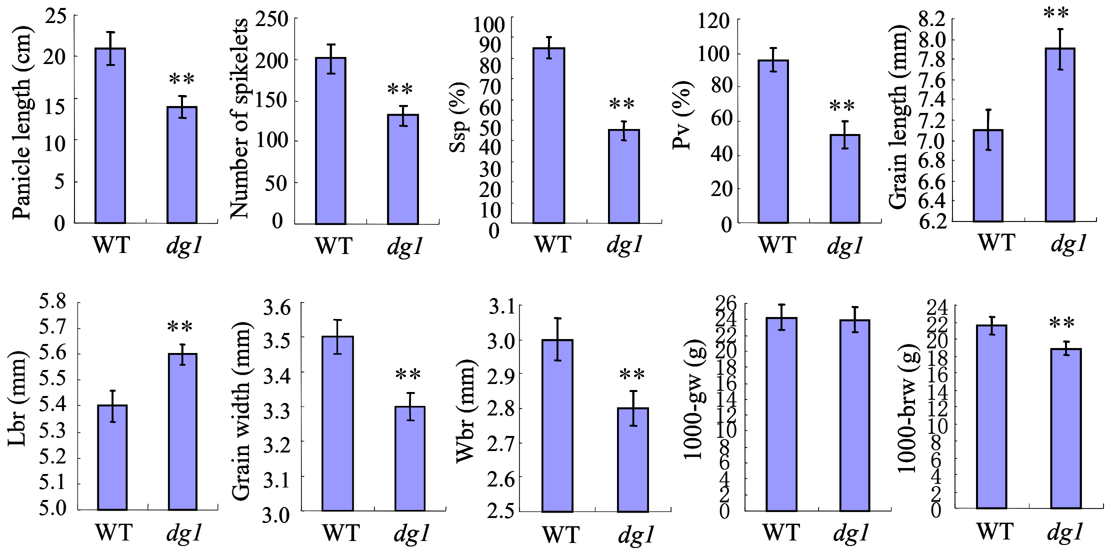

Supplemental Figure 4. Comparison of related traits associated withgrain yield in the wild type and *dg1* mutant. ssp, seed setting percentage, Pv, pollen viability; Lbr, length of brown rice; Wbr, width of brown rice; 1000-gw, 1000-grain weigth; 1000-brw, 1000-weigth of brown rice. **Significant difference at $P<0.01$ compared with the wild type by Student's test.

Supplemental Table

Supplemental Table 1. Primers used in the study.

Purpose	Primer name	Sequence
Mapping	C2-1F	TTGTTGTAAGTTCATGTGGTTGGC
	C2-1R	ATATCTATGGCAATTCTTTGC
	C5-1F	AAGTGGATCCTAAAATTTTGTC
	C5-1R	AATTTCCATACCAACCGAGGG
	C7-1F	TTGCAGATCAAGCTGATGGG
	C7-1R	CCACCTTGAGGAGCTATTCC
	C8-1F	TACAGTCCACAATAATTCAGAGTGATGC
	C8-1R	TTCACCTACCTCCTCCTCTCAACCGAAGA
	C9-1F	ATCTCTCCTTCCAACACATCCATGGCCAC
	C9-1R	ATGGGGGCCCTACCAGACCAGGCCGAGT
	C17-1F	ATCGGGAGCTCCGGCCTGGC
	C17-1R	TACGGCCGCTACCTCGGGACCCCC
	C18-1F	ATCACCCCGGTGATCATGG

	C18-1R	AACAAGTCCTCCTCCGGCGC
	C21-1F	ATCACACGCTATTACCGC
	C21-1R	ATGGAATGATACAGAATCAATGACTGC
	In10-1F	ATTGTCACTAATGAACTCGAGAACG
	In10-1R	TCACTGTTCTTTTGTCCTTGGG
	In11-1F	TTGAAAACTATAGTAACCGG
	In11-1R	AATGAATCTAGACATATATGTGTGC
	In13-1F	TAAATTACAACTCTACAAAAATGG
	In13-1R	TAGCAACGAGAACAAGCTTATCC
	In14-1F	ATAGAAGAGAAAACTCCATC
	In14-1R	TAATCGTTCACCTTGTTTTGCGC
	In15-1F	TAACATTGTGAGATGCAGC
	In15-1R	AAACCTTCCTCTCCACCAACAAGTCC
qRT-PCR	*OsMADS1*-1F	GCTGCAACTACAACTCACAGG
	OsMADS1-1R	TGATGGTGAGCATGAGGGTG
	OsMADS14-1F	CCATTAACGAGCTTCAACGG
	OsMADS14-1R	TGGTATGGATCTGAAGCCTCC
	OsMADS15-1F	AGTACGCCACTGACTCCAGG
	OsMADS15-1R	TGCTGGCCCCTCACATTC
	OsMADS6-1F	CCAACAATGCACTTTCTGAAAC
	OsMADS6-1R	GGAGGCTTGCTGCATGGC
	DL-1F	CCCATCTGCTTACAACCGCTT
	DL-1R	GTTGGAGGTGGAAACCGTCG
	G1-1F	GGCGTCTACTTGCCATTTCTG
	G1-1R	TCGATCAGCATCAAAGCACAG
	OsMADS34-1F	GCTTCGCAAGATGCTGCC
	OsMADS34-1R	GTAGCCAGTGGAGCTAAATCCTC
	SNB-1F	ACCACGAAGTAGGGAACGACTGGG
	SNB-1R	CAGCCAATAAGTCCTCAGTGGCCTG
	OsIDS1-1F	GTCGTCGTCAGTCGAGGCG
	OsIDS1-1R	GCGACTCCACATTGAGATCCA
	MFS1-1F	CGGCTCGTGATCTCGACACGTAC
	MFS1-1R	CACAGCCGGACCAGTGCTCTC
	OsMADS2-1F	GGGATAGTGAACGTGAATGATAAAC
	OsMADS2-1R	GGACATTCAATCCAGTGGTGG
	OsMADS4-1F	CCAATCTGCGGGACAAGA
	OsMADS4 -1R	AGCCAAATTGGCAGTGCTC
	OsMADS16-1F	CCGCTACCAGCAAGCCAT
	OsMADS16-1R	CTTGTAGGTTTCAGTCTGTGTGG
	OsMADS3-1F	GCCAACAACAGTGTGAAATCC
	OsMADS3-1R	TGGCGCAGTTTGGAGGAC
	OsMADS58-1F	TGATGATGCCAGAGCCAGC
	OsMADS58-1R	TCTGTTGCTTCAGCTTAGCAGCT
	BG1-1F	GATGGAGAGCGACGAGGAC

BG1-1R	GCAATGGCGGCGAAGTTC
BG2-1F	GACGTTGTCGTGCTCGCATC
BG2-1R	TCGCCTACACCCCTAAGAGC
DSG1-1F	AGCCATGGCGGGAGGTT
DSG1-1R	CGGTGACCTCGAACACGTT
FBK12-1F	GGATCACTCTTGGTAAGTTGCC
FBK12-1R	CGATGACAATCAACCGATCAC
FUWA-1F	AGCAACATTGTGCGAATAACTCC
FUWA-1R	TTCCTGTATCATCCACGGCAA
GIF-1F	CATGTACCAGCCGACGTTTG
GIF-1R	GCTCTCAACAACCGACCTGTC
GL3-1F	GCTCAAGGTCACCTGATCACTC
GL3-1R	GAACGACCACAAGATCTCTGC
GL7-1F	CCCCTAGCATCGACACCAAG
GL7-1R	CGGGTTCCAGCACTCCTCT
GS2-1F	TGCGTCCCTTCTTTGATGAGT
GS2-1R	ACAGTTGGGTGCCTGAGAATG
GS3-1F	CGGAAGAACTCCTGATCCATTC
GS3-1R	CACTTGCTCTGCACAAACAGC
GS5-1F	GTTCTCGGTACTGCGTGGAAG
GS5-1R	ACTCCACAAACCTCCCAGCA
GW2-1F	CAGCCACCCAGTATGGACTTC
GW2-1R	ACATGCTTCCACCAGCAATGT
GW8-1F	GGGATGATCAAAACCGAGGAG
GW8-1R	GTCAGAGGTGGAGCCAACGA
TGW6-1F	GCCACAACGAGAATGTTCAAG
TGW6-1R	CAGCTCATTAGGGTGCAGATGAC
CYCD3-1F	CCTTCCACACTGACGGTACAGTT
CYCD3-1R	TGCCGCTGCCAAATAGACA
CAK1A-1F	GACCGACAAGGGTTTCAGCAT
CAK1A-1R	CCAGCATGTTCAGGAAGATACAAT
CDKA-1F	GGTTTGGACCTTCTCTCTAAAATGC
CDKA-1R	AGAGCCTGTCTAGCTGTGATCCTT
CYCT1-1F	GCATTTGTTGCAGCTCAAG
CYCT1-1R	TCACCACTTCGCTGACTTATTG
MCM4-1F	CCCGAATGCGATTCTCTGAA
MCM4-1R	ACCAGTGGCATGATCAGTTGC
MCM5-1F	AAGGAGAACTGCCTGTCCATGA
MCM5-1R	AGTGGCCTTAGCTTTCACCCTC
CYCB2.1-1F	AAGTTTGGCCAGGAGTGAGCA
CYCB2.1-1R	TCAAGAGCATCAGCGTCGAGA
CDC20-1F	TCGAATCACCTGTTTGTTGGC
CDC20-1R	TGGAGACAATCCAACGCAAAG
OsEXPA2-1F	TTTGGCTATTCTGAGGCTGCT

OsEXPA2-1R	TGGTCCCAAAAGCACAAGAGT
OsEXPA32-1F	GCAGTTCGGCGTCACCTACCAG
OsEXPA32-1R	GGTTCGATCCAGATTTGCAGTAGTCA
OsEXPB5-1F	TGTTTGTTAACGTCGCCGCGATAG
OsEXPB5-1R	TCACTAGAAGCAGCTCTGCAAACG
ACTIN-F	GACCCAGATCATGTTTGAGACCT
ACTIN-R	CAGTGTGGCTGACACCATCAC

Supplemental Table 2

	rg (mm)	sl (mm)	le (mm)	pa (mm)
WT	0.5±0.1	2.0±0.3	7.1±0.4	6.5±0.3
dg1	1.1-4.0	6.0-7.9	7.9±0.3	7.4±0.4

Supplemental Table 2. Organ size in the wild type and *dg1* mutant. rg, rudimentary glume; sl, sterile lemma; le, lemma; pa, palea.
